# Ethanol-Induced Behavioral Sensitization Alters the Synaptic Transcriptome and Exon Utilization in DBA/2J Mice

**DOI:** 10.3389/fgene.2018.00402

**Published:** 2018-09-24

**Authors:** Megan A. O’Brien, Rory M. Weston, Nihar U. Sheth, Steven Bradley, John Bigbee, Ashutosh Pandey, Robert W. Williams, Jennifer T. Wolstenholme, Michael F. Miles

**Affiliations:** ^1^Department of Pharmacology and Toxicology, Virginia Commonwealth University, Richmond, VA, United States; ^2^VCU Alcohol Research Center, Virginia Commonwealth University, Richmond, VA, United States; ^3^Department of Anatomy and Neurobiology, Virginia Commonwealth University, Richmond, VA, United States; ^4^Department of Genetics, Genomics and Informatics, The University of Tennessee Health Science Center, Memphis, TN, United States; ^5^Department of Neurology, Virginia Commonwealth University, Richmond, VA, United States

**Keywords:** synaptic, mRNA trafficking, RNAseq, exon utilization, ethanol, sensitization

## Abstract

Alcoholism is a complex behavioral disorder characterized by loss of control in limiting intake, and progressive compulsion to seek and consume ethanol. Prior studies have suggested that the characteristic behaviors associated with escalation of drug use are caused, at least in part, by ethanol-evoked changes in gene expression affecting synaptic plasticity. Implicit in this hypothesis is a dependence on new protein synthesis and remodeling at the synapse. It is well established that mRNA can be transported to distal dendritic processes, where it can undergo localized translation. It is unknown whether such modulation of the synaptic transcriptome might contribute to ethanol-induced synaptic plasticity. Using ethanol-induced behavioral sensitization as a model of neuroplasticity, we investigated whether repeated exposure to ethanol altered the synaptic transcriptome, contributing to mechanisms underlying subsequent increases in ethanol-evoked locomotor activity. RNAseq profiling of DBA/2J mice subjected to acute ethanol or ethanol-induced behavioral sensitization was performed on frontal pole synaptoneurosomes to enrich for synaptic mRNA. Genomic profiling showed distinct functional classes of mRNA enriched in the synaptic vs. cytosolic fractions, consistent with their role in synaptic function. Ethanol sensitization regulated more than twice the number of synaptic localized genes compared to acute ethanol exposure. Synaptic biological processes selectively perturbed by ethanol sensitization included protein folding and modification as well as and mitochondrial respiratory function, suggesting repeated ethanol exposure alters synaptic energy production and the processing of newly translated proteins. Additionally, marked differential exon usage followed ethanol sensitization in both synaptic and non-synaptic cellular fractions, with little to no perturbation following acute ethanol exposure. Altered synaptic exon usage following ethanol sensitization strongly affected genes related to RNA processing and stability, translational regulation, and synaptic function. These genes were also enriched for targets of the FMRP RNA-binding protein and contained consensus sequence motifs related to other known RNA binding proteins, suggesting that ethanol sensitization altered selective mRNA trafficking mechanisms. This study provides a foundation for investigating the role of ethanol in modifying the synaptic transcriptome and inducing changes in synaptic plasticity.

## Introduction

Alcoholism is a chronic disease characterized by compulsive drug-seeking undeterred by negative consequences, as well as cravings and potential for relapse that persist despite years of abstinence. The endurance of these pernicious behaviors supports the theory that addiction arises from progressive and lasting cellular and molecular adaptations in response to repeated ethanol exposure (Nestler et al., 1993; Nestler, 2001). A more complete comprehension of neuronal plasticity that underlies the transition to compulsive drug use could lead to novel therapeutic strategies for alcohol use disorders.

The morphological specialization of neurons, where synapses appear to be regulated in an individual manner, advocates the need for local mechanisms controlling synaptic function. Local synaptic protein synthesis is supported by the finding of synthesis machinery at post-synaptic sites, including ribosomes, tRNA, translation factors, endoplasmic reticulum, and Golgi apparatus (Steward and Levy, 1982; Steward and Reeves, 1988). Furthermore, through *in situ* hybridization (Lyford et al., 1995; Poon et al., 2006) and studies characterizing synapse-enriched subcellular fractions (Chicurel et al., 1993; Rao and Steward, 1993; Poon et al., 2006; Matsumoto et al., 2007) and microdissected neuropil (Cajigas et al., 2012), a number of mRNA species have been identified at synapses. mRNA transport has been shown to occur in an activity dependent manner. For instance, mRNA of the immediate early gene, *Arc*, as well as *GluR1* and *GluR2* transcripts have been shown to be localized to dendrites following NMDA and metabotropic glutamate receptor activation, respectively (Steward and Worley, 2001; Grooms et al., 2006). Also, depolarization extends transport of mRNA for BDNF and its receptor, TrkB, to the distal processes in neuronal cell culture (Tongiorgi et al., 1997). Studies using protein synthesis inhibitors have shown that protein synthesis is required for behavioral and synaptic plasticity, assumedly for establishing enduring modifications (Kang and Schuman, 1996; Steward and Schuman, 2001). Thus, targeting of specific RNAs to dendrites may be an efficient way of rapidly localizing proteins involved in synaptic function. Alterations in dendritic mRNA transport, stability, or translation could thus modulate synaptic plasticity (Steward and Banker, 1992; Chicurel et al., 1993).

Previous research from our laboratory that examined ethanol regulation of gene expression across a variety of mouse strains has found significant enrichment of genes involved with synaptic functioning and plasticity, reproducibly among several brain regions (Kerns et al., 2005; Wolen et al., 2012). There is also evidence to support that adaptive responses underlying ethanol tolerance and dependence are synaptic in nature, in part involving changes in glutamate neurotransmission (Tsai and Coyle, 1998). Ethanol administration has been shown to induce structural synaptic plasticity as well. Alcohol-preferring rats exposed to 14 weeks of continuous access or subjected to repeated deprivations of ethanol exhibited decreased density and increased size of spines in a subpopulation of neurons in the nucleus accumbens (Zhou et al., 2007). Cortical neurons exposed to chronic intermittent ethanol administration had significant increases in NMDA receptor surface expression (Qiang et al., 2007) and hippocampal cultures receiving prolonged ethanol exposures exhibited increased co-localization of PSD95 and f-actin (Carpenter-Hyland and Chandler, 2006) leading to enlargement of spine heads. Together these data suggest that dendritic spines may be an important target for the adaptive actions of ethanol. Therefore, we investigated whether ethanol evoked changes to the synaptic transcriptome in a well-characterized model of behavioral plasticity, ethanol locomotor sensitization.

It has been proposed that behavioral sensitization is a process that occurs following repeated drug exposure as the result of neuroadaptations in brain reward systems that contribute to such phenomenon as drug craving and relapse in alcoholics (Piazza et al., 1990; Robinson and Berridge, 1993). Intermittent administration of many drugs of abuse, including ethanol, propagates the development of long-lasting sensitized responses to their stimulant effects, often measured as augmented locomotor activation in rodent models (Shuster et al., 1975; Hirabayashi and Alam, 1981; Masur et al., 1986). Behavioral sensitization has been associated with neurochemical and molecular adaptions that effect neurotransmission (Kalivas and Stewart, 1991; White and Kalivas, 1998; Vanderschuren and Kalivas, 2000). There is also evidence that brain regions mediating reinforcement and reward undergo neuroadaptations with cocaine or amphetamine sensitization causing increased incentive salience and self-administration of the drug (Horger et al., 1990; Piazza et al., 1990). Increased voluntary consumption of ethanol has also been observed following intermittent repeated exposure (Lessov et al., 2001; Camarini and Hodge, 2004).

We therefore hypothesize that ethanol-induced sensitization may result, at least in part, from alterations in the synaptic transcriptome, contributing to synaptic remodeling and plasticity. Here we utilize synaptoneurosomes (Williams et al., 2009) prepared from ethanol sensitized DBA2/J mice to enrich for synaptic mRNAs for the purpose of RNAseq analysis. Our expression profiling reveals that repeated ethanol exposure elicits distinctive changes to the complement of mRNA present at the synapse. Furthermore, our detailed analysis identifies, for the first time, that ethanol behavioral sensitization produces a striking alteration in exon utilization in the synaptic compartment. This analysis of the synaptic transcriptome in response to ethanol sensitization increases our understanding of mechanisms underlying ethanol-induced synaptic plasticity and highlights the complexity of genomic regulation at the subcellular level.

## Materials and Methods

### Ethics Statement

All procedures were approved by Virginia Commonwealth University Institutional Animal Care and Use Committee under protocol AM10332 and followed the NIH Guide for the Care and Use of Laboratory Animals (NIH Publications No. 80-23, 1996).

### Animals

Male DBA/2J (D2) mice were purchased from Jackson Laboratories (Bar Harbor, ME) at 8–9 weeks of age. Animals were housed four per cage and had *ad libitum* access to standard rodent chow (#7912, Harlan Teklad, Madison, WI, United States) and water in a 12-h light/dark cycle (6 am on, 6 pm off). Mice were housed with Teklad corn cob bedding (#7092, Harlan Teklad, Madison, WI, United States) and cages were changed weekly. Subjects were allowed to habituate to the animal facility for 1 week prior to starting behavioral experiments. Behavioral assays were performed during the light cycle between the hours of 8 am and 2 pm.

### Ethanol-Induced Behavioral Sensitization and Tissue Collection

Ethanol (EtOH) behavioral sensitization was induced as previously described (Costin et al., 2013a,b). Briefly, mice were divided into three treatment groups (*n* = 16 each): saline–saline (SS), saline–EtOH (SE), and EtOH–EtOH (EE). Mice were acclimated to the behavioral room for 1 hour prior to the start of the experiment on testing days. All locomotor activity was measured immediately following i.p. injection with either saline or ethanol during 10-min sessions in sound-attenuating locomotor chambers (Med Associates, model ENV-515, St. Albans, VT, United States). The system is interfaced with Med Associates software that assesses activity using a set of 16 infrared beam sensors along the *X*–*Y* plane. Animals received 2 days of saline injections and placement in the testing apparatus for habituation to the experimental procedure. On test day 3, acute locomotor responses to i.p. saline (SS, SE) or 2.0 g/kg ethanol (EE) were measured. On conditioning days 4–13, animals received daily i.p. injections in their home cages of either saline (SS, SE) or 2.5 g/kg ethanol (EE). On the final testing day 14, the SS group received saline and the SE and EE groups received 2.0 g/kg ethanol and all groups were subsequently monitored in activity chambers for 10 min. On day 14 of the behavioral sensitization paradigm, mice were sacrificed by cervical dislocation 4 h following i.p. injection. Immediately afterward, brains were removed and chilled for one minute in ice-cold 1x phosphate buffered saline. The frontal pole was dissected by making a cut rostral of the optic chiasm and then removing the olfactory bulbs. Excised tissue was stored in a tube on ice for less than 8 min before processing for synaptoneurosome isolation.

### Synaptoneurosome Preparation

The protocol for preparation of synaptoneurosomes was adapted from Williams et al. (2009). Fresh tissue from four animals was pooled (approximately 0.45 g) and manually homogenized utilizing a 15 mL Potter-Elvehjem Safe-Grind^®^ tissue grinder (#358009, Wheaton, Millville, NJ, United States) and diluted 1:10 in synaptoneurosome homogenization buffer. The buffer consisted of 0.35 M nuclease free sucrose (CAS #57-50-1, Acros Organics, NJ), 10 mM HEPES (#15630-056, Life Technologies, Carlsbad, CA, United States), and 1 mM EDTA (#AM9260G, Ambion, Carlsbad, CA, United States), which was brought to a pH of 7.4 and filter sterilized. Immediately before use, 0.25 mM DTT (CAS #3483-12-3, Fisher Scientific, Waltham, MA, United States), 30 U/mL RNase Out (#10777-019, Invitrogen, Carlsbad, CA, United States), and protease inhibitor cocktail containing AEBSF, aprotinin, bestatin, E64, leupeptin, and pepstatin A (#1862209, Halt, Thermo Scientific, Rockford, IL, United States) were added to buffer. Centrifugation of whole homogenate (WH) at 500 ×*g* for 10 min at 4°C removed nuclei and cellular debris, yielding pellet, P1 and supernatant, S1. The S1 fraction was passed through a series of nylon filters with successively decreasing pore sizes of 70, 35, and 10 μm (#03-70, #03-35, #03-10, SEFAR, Buffalo, NY). The filtrate was then diluted with 3 volumes of homogenization buffer and centrifuged at 2000 ×*g* for 15 min at 4°C to yield the synaptoneurosome enriched pellet, P2, and a cellular supernatant fraction, S2. Fractions were frozen on dry ice and then stored at -80°C until further processing. Aliquots from each fraction of a synaptoneurosomal preparation were examined for the presence of contaminating nuclei using 4′,6-diamidino-2-phenylindole (DAPI) staining. Representative fields at 20x magnification were assessed for nuclear content.

### Transmission Electron Microscopy (TEM)

Morphological integrity of synaptoneurosomes was confirmed by transmission electron microscopy (TEM). The P2 fraction was washed in PBS and centrifuged at 2000 ×*g* for 8 min. The supernatant was decanted and pellet was fixed with 2% glutaraldehyde in 0.1 M sodium cacodylate buffer at room temperature. After initial fixation, the sample was rinsed in 0.1 M cacodylate buffer for 5–10 min and then post-fixed in 1% osmium tetroxide in 0.1 M cacodylate buffer for 1 h, followed by another 5–10 min rinse in 0.1 M cacodylate buffer. Preparation continued with a serial dehydration with ethanol: 50, 70, 80, and 95% – for 5–10 min each, followed by 100% ethanol for 10–15 min (3x), and incubation in propylene oxide for 10–15 min (3x). The sample was then infiltrated with a 50/50 mix of propylene oxide and PolyBed 812 resin (Polysciences, Inc., Warrington, PA, United States) overnight, which was then replaced with pure resin once again overnight. The sample was embedded in a mold, placed in a 60°C oven overnight, and then sectioned with a Leica EM UC6i Ultramicrome (Leica Microsystems, Wetzlar, Germany), stained with 5% Uranyl acetate and Reynold’s Lead Citrate, and examined on JEOL JEM-1230 transmission electron microscope (JEOL USA, Inc., Peabody, MA, United States). Images of various magnifications (2000x–10,000x) were captured with the Gatan Ultrascan 4000 digital camera (Gatan, Inc., Pleasanton, CA, United States).

### Immunoblotting

Pellets (P1 and P2) and liquid aliquots (WH, S1, and S2) from synaptoneurosomal preparations were used to perform semi-quantitative immunoblotting. Pellets were triturated with NuPAGE LDS (#NP0008, Life Technologies, Carlsbad, CA, United States) diluted to 1x and containing protease inhibitor cocktail (#1862209, Halt, Thermo Scientific, Rockford, IL, United States), while liquid aliquots were lysed directly with 4x LDS with added proteinase inhibitor. Samples were sonicated on ice water until no longer viscous. Protein concentrations were determined using the bicinchoninic acid assay (#23227, Thermo Scientific, Rockford, IL, United States) and absorbance at 562 nm. Sample concentrations were balanced using 1x LDS, 10x NuPAGE reducing agent (#NP0004, Life Technologies, Carlsbad, CA, United States) and boiled for 10 min. For each synaptoneurosome fraction, 10 μg of protein was loaded per lane on a 10% or a 4–12% NuPAGE bis-tris gel (#NP0303BOX, #NP0322BOX, Life Technologies, Carlsbad, CA, United States). Electrophoresis was performed at 150 V followed by transfer to 0.45 μm nitrocellulose membrane for 1.5 h at 30 V on ice. Membranes were incubated with Ponceau S for 10 min, and densitometric analysis of staining was performed using ImageJ processing and analysis software (National Institutes of Health). Prior to primary antibody incubation, the membranes were blocked with 5% non-fat dried milk in 1x TBST for 45 min. Primary and secondary antibody catalog numbers, dilutions, and incubation times are provided in **Supplementary Table S1**. Immunoblots were visualized on GeneMate Blue Autoradiography film (BioExpress, Kaysville, UT, United States) using the Amersham ECL Western Blotting Detection Reagent (#RPN2106, GE Healthcare Life Sciences, Pittsburgh, PA, United States) and quantified using ImageJ. All detected proteins were normalized to the total protein loaded per well as measured by Ponceau S staining. Statistical analysis of immunoblot data was performed by one-way ANOVA across synaptoneurosome fractions followed by Tukey’s *post hoc* analysis.

### Quantitative Reverse Transcriptase PCR (qRT-PCR)

Synaptoneurosomal fractions, S2 and P2, prepared from mice subjected to the sensitization protocol were assessed for enrichment of known dendritically-trafficked and somatically-restricted transcripts using qRT-PCR. Total RNA was isolated the using guanidine/phenol/chloroform method (#Cs-502, Stat-60, Tel-Test Inc., Friendswood, TX, United States) and a Tekmar homogenizer as per the STAT-60 protocol. RNA concentration was determined by measuring absorbance at 260 nm and RNA quality was assessed by electrophoresis on an Experion Analyzer (Bio-Rad, Hercules, CA, United States) and 260/280 absorbance ratios. All RNA samples had RNA quality indices (RQI) ≥ 7.6 and 260/280 ratios were between 1.97 and 2.06. cDNA was generated from 995 ng of total DNase-treated RNA and 5 ng of luciferase mRNA (#L4561, Promega, Madison, WI, United States) using Deoxyribonuclease I (#18068-015, Invitrogen, Carlsbad, CA, United States) and the iScript cDNA kit (#170-8891, Bio-Rad, Hercules, CA, United States) according to manufacturer’s instructions. qRT-PCR was performed using the iCycler iQ system (Bio-Rad, Hercules, CA, United States) according to manufacturer’s instructions for iQ SYBR Green Supermix (#170-8880, Bio-Rad, Hercules, CA, United States). Primer sequences, annealing temperatures, amplicon sizes, and cDNA dilutions used for each gene are listed in **Supplementary Table S1**. Relative expression was calculated by comparing Ct values to a standard curve produced from S2 fraction cDNA (diluted 1:5, 1:25, 1:125, 1:625). Expression values were normalized to the exogenous internal reference mRNA, luciferase, to control for losses and inefficacies of downstream processing (Johnson et al., 2005). Statistical analysis of qRT-PCR data was performed using a Student’s *t*-test between the two fractions.

### RNAseq Library Preparation and Sequencing

**FIGURE 1:**
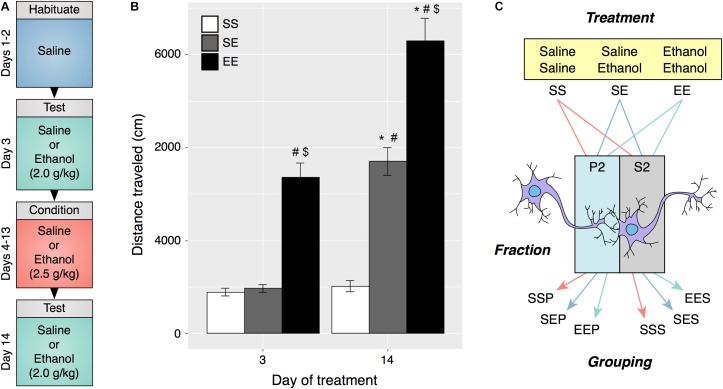
Ethanol behavioral sensitization in male DBA2/J mice. **(A)** Experimental protocol and timeline for induction of behavioral sensitization. **(B)** Repeated ethanol exposure induced behavioral sensitization as measured by locomotor activity on day 14 (EE) as compared to acute ethanol administered on day 3 (EE) and day 14 (SE). **(C)** Experimental groupings used for RNA sequencing and bioinformatic analysis were derived from ethanol treatment type and specific cellular fraction. (#*p* < 0.001 compared to SS within same day, $*p* < 0.001 compared to SE within same day, ^∗^*p* < 0.001 compared to same treatment on day 3, repeated measures two-way ANOVA with Tukey’s *post hoc* analysis).

RNAseq data have been deposited with the Gene Expression Omnibus resource (GSE73018). Total RNA isolated for qRT-PCR was also used for gene expression profiling using RNAseq performed by the VCU Genomics Core Laboratory. To avoid non-biological experimental variation that arises from sample batch structure, supervised randomization of samples prior to each processing stage (RNA extraction, library amplification, and lane assignment) was performed. A total of four biological replicates, each representing a pool from four animals, were obtained for each treatment group/fraction (**Figure 1**; SSS, SES, EES, SSP, SEP, EEP). Preparation of cDNA libraries was conducted following standard protocols using TruSeq RNA Sample Preparation Kit (#RS-122-2001, Illumina, San Diego, CA, United States). Briefly, mRNA was isolated from total RNA using poly-T oligo-attached magnetic beads and then fragmented in the presence of divalent cations at 94°C. Fragmented RNA was converted into double stranded cDNA followed by ligation of Illumina specific adaptors. Adaptor ligated DNA was amplified with 15 cycles of PCR and purified using QIAquick PCR Purification Kit (#28104, Qiagen, Venlo, Netherlands). Library insert size was determined using an Agilent Bioanalyzer. Library quantification was performed by qRT-PCR assay using KAPA Library Quant Kit (#KK4835, KAPA, Wilmington, MA, United States). RNAseq libraries were analyzed using Illumina TruSeq Cluster V3 flow cells and TruSeq SBS Kit V3 (#FC-401-3001, Illumina, San Diego, CA, United States), with six libraries of different indices pooled together in equal amounts loaded on to a single lane at a concentration of 13 pM and sequenced (2 × 100 paired end reads) on an Illumina HiSeq 2000. Sample EE6_P2 was removed from subsequent analyses due to over-amplification artifacts. A summary of RNAseq metrics can be found in **Supplementary Table S2**.

### RNAseq Alignment

FASTQ formatted sequence files were aligned using TopHat2 v2.0.8 (Kim et al., 2013) with GRCm38/mm10 reference genome and annotations obtained from the UCSC genome table browser^1^ (Karolchik, 2004). The C57BL/6 (B6) reference genome (mm10) was edited to include DBA2/J (D2) single nucleotide polymorphisms (Wang et al., 2016). Aligned BAM files produced by TopHat2 were validated for mapping quality with Samtools v0.1.9 (Li et al., 2009) and for completeness using BamUtil v1.0.13^2^. BAM files were converted to sorted SAM files for downstream feature count-based analysis with Samtools.

### Differential Gene Expression Analysis

Raw read counts were produced from each SAM file using the python package HTSeq v0.6.1 (Anders et al., 2012) script *htseq-count* with the read overlap handler set to *union*. Resulting raw count files were analyzed for differential gene expression (DGE) between ethanol sensitized (EE) or acutely exposed (SE) animals and ethanol naïve (SS) animals within either the synaptic P2 fractions or the cellular supernatant S2 fractions using the R^3^ package edgeR v3.10.2 (Robinson et al., 2010) with a negative binomial generalized log-linear model approach (Mccarthy et al., 2012). Lowly expressed genes were filtered out if not present in at least three libraries with counts per million of 3.4 or greater, corresponding with approximately five total counts in the smallest library. Genes meeting a false discovery rate (FDR) cutoff of 0.10 were considered significantly altered and used in downstream bioinformatic analysis.

### Differential Exon Usage Analysis

A GFF annotation file containing collapsed exon counting bins was prepared from the UCSC GRCm38/mm10 GTF file using the DEXSeq v1.16.10 (Anders et al., 2012) Python script *dexseq_prepare_annotation.py* with gene aggregation disabled. The number of reads overlapping each exon bin was then counted using the DEXSeq Python script *dexseq_count.py*, the GFF file, and each sample’s SAM file. Differential exon usage (DEU) analysis was then carried out for the same contrasts studied in our DGE analysis using the DEXSeq R package standard analysis workflow. Ensembl transcript IDs produced in the DEXSeq results files were translated to gene symbols using the R package BiomaRt v2.32.0 (Durinck et al., 2009). Genes with transcripts possessing at least one differentially utilized exon bin with an adjusted *p*-value (*p*adj) less than 0.01 were considered to be significantly altered and were used in downstream bioinformatic analysis.

### Bioinformatic Analysis

Functional enrichment analyses for DGE and DEU results were performed using ToppFun, available as part of the ToppGene suite of web-based applications^4^ (Chen et al., 2009). Mouse gene symbols were submitted and analyzed for over-representation of genes that belong to Gene Ontology categories (molecular function, biological processes, and cellular component), mouse phenotypes, and biological pathway databases including KEGG and Reactome. Only categories with *p*-values less than 0.01 and possessing between 3 and 1000 total genes were considered. The webtool REVIGO (Supek et al., 2011) was used for data reduction by semantic similarity, and visualization of GO terms lists resulting from this analysis.

### RNA Binding Protein Enrichment Analysis

Genes possessing DEU between EEP and SSP groups (*p*adj < 0.01) were intersected with the genes possessing basal DEU between SSP and SSS groups (*p*adj < 0.01) in order to produce a list of genes with synapse-specific DEU that was also regulated by ethanol sensitization. The same was done to produce a synaptic sensitization-induced DGE gene list using FDR cutoffs of 0.1. These two lists of genes were then intersected with gene list obtained from two public databases of known and predicted RNA binding proteins (RBPs): RBPDB (Cook et al., 2011) and ATtRACT (Giudice et al., 2016). The synaptic ethanol-sensitive DEU gene list was also intersected with a list of mRNA targets of the RBP fragile X mental retardation protein (FMRP), which was obtained from **Supplementary Table S2a** of Darnell et al. (2011). For RNABP and FMRP enrichment analyses, the R package GeneOverlap (version 1.16.0^5^) was used to calculate odds ratios for relative enrichment of synaptic ethanol sensitive DEU genes and Fisher’s exact tests to calculate enrichment *p*-values.

### Sequence Motif Analysis

Chromosomal coordinates for the differentially utilized exon bins from the synaptic sensitization-induced DEU gene lists used in the RNABP analysis were provided to BEDTools v2.26.0 (Quinlan and Hall, 2010) in order to obtain their respective nucleotide sequences. Sequences for the 475 exon bins (**Supplementary Table S12**) containing a minimum of eight base pairs were then supplied to the web-based motif discovery tool MEME (Bailey et al., 2009) to search for known or novel motifs common between them. Any motifs identified that met an *E*-value cutoff of 0.05 were aligned to the CISBP-RNA database of RNABP motifs and specificities using the MEME Suite tool Tomtom (Gupta et al., 2007). Database motif alignments were considered significant if the alignment score had an *E*-value ≤ 0.05.

## Results

### Synaptoneurosome Fractions Are Enriched in mRNA Coding Synaptic Components

DBA/2J (D2) mice were chosen for these studies due to their characteristic sensitivity to ethanol psychomotor stimulation and development of sensitization (Phillips et al., 1994). Distance traveled on test days 3 and 14 was compared and a significant increase in activity on day 14 was interpreted as an induction of ethanol sensitization (**Figures 1A,B**). Daily i.p. injections of 2.5 g/kg ethanol elicited an augmented locomotor response to 2.0 g/kg ethanol on day 14 as compared to day 3 (two-way repeated measures ANOVA, *F*_Treatment_[2,45] = 96.76, *p* < 0.001, *F*_Day_[1.45] = 77.47, *p* < 0.001, *F*_Interaction_[2,45] = 16.89, *p* < 0.001, *n* = 16). Frontal pole brain tissue obtained from mice in this experiment was utilized in preparation of synaptoneurosome enriched samples.

**FIGURE 2:**
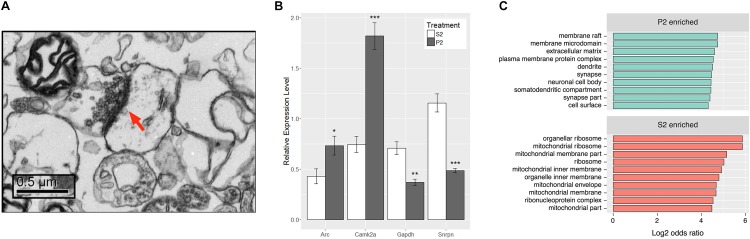
Synaptoneurosome preparation produces distinct RNA populations between P2 and S2 fractions. **(A)** Representative electron micrograph from P2 fraction observed at 10,000x magnification. Post-synaptic density is labeled by red arrow and presynaptic elements with synaptic vesicles can be observed immediately adjacent. **(B)** RNA isolated from S2 and P2 fractions of behaviorally sensitized mice was assayed for transcripts of known subcellular localization to ensure enrichment of synaptic RNAs. *Camk2a* and A*rc* are transcripts known to be synaptically targeted, while *Gapdh* and *Snrpn* are somatically restricted. Paired Student’s *t*-test between fraction for each gene, *Camk2a* (*t*[7] = 6.941, ^∗∗∗^*p* = 0.0002), *Arc* (*t*[7] = 2.646, ^∗^*p* = 0.0331), *Gapdh* (*t*[7] = 4.181, ^∗∗^*p* = 0.0041), *Snrpn* (*t*[7] = 8.439, ^∗∗∗∗^*p* < 0.0001), *n* = 8. **(C)** Top 10 Gene Ontology Cellular Compartment categories according to *p*-value as derived from functional enrichment analysis of the untreated P2 enriched gene list (SSP vs. SSS), sorted by log_2_ of the categories’ odds ratio.

The synaptoneurosomal fractionation protocol (**Supplementary Figure S1A**) was validated in preliminary studies by TEM (**Figure 2A**). As suggested previously (Williams et al., 2009), the intact pre- and post-synaptic terminals, identified by TEM, provide for selective extraction of synaptic mRNAs. Absence of intact nuclei throughout synaptoneurosomal fractions was verified by DAPI staining (**Supplementary Figure S1B**), while immunoblotting for subcellular protein markers was used to ascertain purity of the preparation (**Supplementary Figure S1C**). Together these data indicate that P2 fractions contain synaptic elements enriched for the synaptic protein markers, synaptotagmin and PSD-95 (one-way ANOVA, *F*_SY T_[4,10] = 9.83, *p* = 0.0017, *F*_PSD95_[4,10] = 11.09, *p* = 0.0011, *n* = 3), and are devoid of appreciable nuclear contamination (one-way ANOVA, *F*_H4_[4,10] = 125.3, *p* < 0.0001, *n* = 3),

To ensure enrichment in experimental tissues, total RNA isolated from S2 and P2 fractions of mice subjected to the ethanol behavioral sensitization paradigm was evaluated by qRT-PCR (**Figure 2B**). P2 fractions had higher relative expression levels of known synaptically targeted transcripts, *CamK2a* and *Arc* (Burgin et al., 1990; Link et al., 1995; Lyford et al., 1995), while transcripts known to be somatically restricted, *Gapdh* and *Snrpn* (Litman et al., 1994; Poon et al., 2006), were more abundant in the S2 fraction (Student’s paired *t*-test, *t_CamK2a_*[7] = 6.941, *p* = 0.0002, *t_Arc_*[7] = 2.646, *p* = 0.0331, *t_Gapdh_*[7] = 4.181, *p* = 0.0041, *t_Snrpn_*[7] = 8.439, *p* < 0.0001, *n* = 8).

RNAseq was used to evaluate global gene expression in the S2 and P2 fractions (**Supplementary Table S3**). DGE analysis (**Supplementary Tables S4**, **S5**) demonstrated widespread and highly significant differences in P2 vs. S2 samples at the gene level in saline control samples (SSP_SSS), with 1829 genes differentially expressed at an FDR ≤ 0.1 and log_2_ fold-change ≥ 1 or ≤-1. Of these, 1408 were found to be enriched (>twofold increased expression) in the P2 fraction (**Supplementary Table S5**) and 421 enriched in the S2 fraction (**Supplementary Table S5**). Of note, our RNAseq data faithfully replicated the qRT-PCR results of **Figure 2B**, even though derived from a totally separate experiment and synaptoneurosome preparation (**Supplementary Table S6**). This supports the rigor of our RNAseq studies. Functional enrichment analysis of the P2 enriched gene list revealed significant over-representation of cellular categories related to the structure of the synapse (**Figure 2C**) and molecular or biological categories relating to calcium ion binding, cell adhesion, and growth factor binding among others relevant to the synapse (**Supplementary Table S5**). In contrast, the S2 fraction showed cellular category enrichment relating to protein synthesis and mitochondria (**Supplementary Table S5**). These results establish that, in contrast to the cellular supernatant S2 fraction, the P2 synaptoneurosome fraction was enriched for mRNA relevant to synaptic function.

### Sensitizing Ethanol Treatment Alters the Synaptic Transcriptome

**FIGURE 3:**
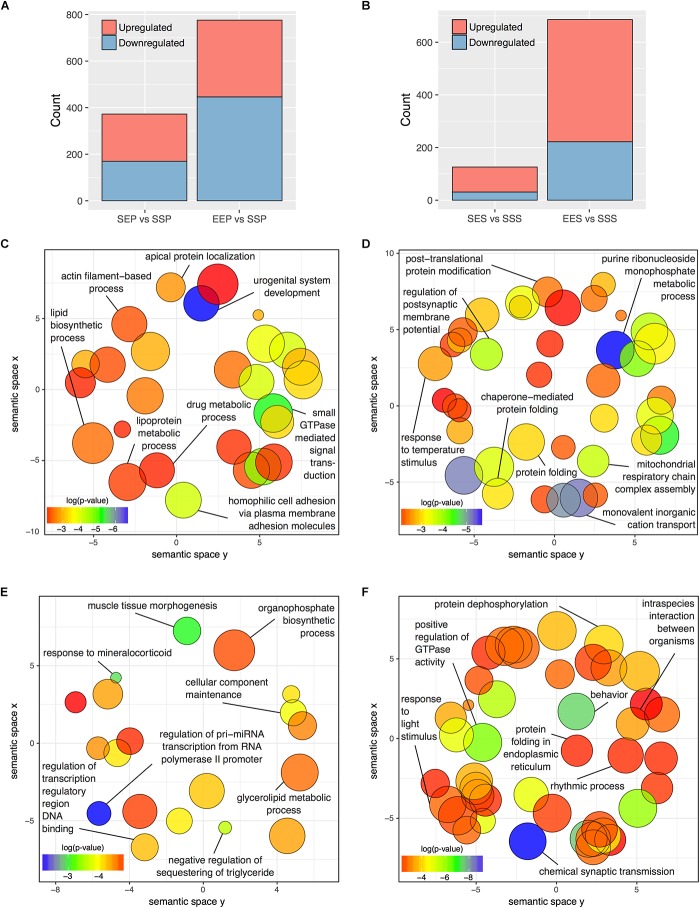
DGE in P2 and S2 fractions following acute ethanol exposure or sensitization. The number of genes found to be significantly altered (FDR < 0.1) by sensitization and acute exposure to ethanol treatments in the **(A)** P2 fraction and **(B)** S2 fractions. Scatterplots of representative Gene Ontology Biological Process categories derived from functional enrichment analysis of genes regulated by acute ethanol **(C,E)** or ethanol sensitization **(D,F)** in the P2 fraction **(C,D)** and S2 fraction **(E,F)**. Scatterplots depict semantic similarity on axes, dispensability by size, and log_10_ *p*-value as color.

To focus our attention on functional reorganization of the synapse occurring with acute ethanol or ethanol sensitization, we identified treatment-responsive DGE within cellular fractions through gene-level analyses in edgR. For these analyses, we used only an FDR cutoff (≤0.1) without further filtering for fold-change. **Figure 3A** and **Supplementary Table S7** show that more than twice as many genes responded to ethanol sensitization (EEP vs. SSP; *n* = 776) as to acute ethanol (SEP vs. SSP; *n* = 375) in the P2 fraction. The S2 fraction (**Figure 3B** and **Supplementary Table S8**) showed an even larger divergence between acute and repeated ethanol exposures with 686 genes regulated by sensitization (EES vs. SSS) and 126 responding to acute ethanol (SES vs. SSS).

Functional over-representation analysis of these DGE groups showed striking divergence between responses to acute vs. sensitizing ethanol treatments within both the P2 and S2 compartments. REVIGO semantic similarity analysis was used to group similar Gene Ontology Biological Process categories and thus reduce the complexity of the functional group analysis. **Figure 3D** demonstrates functional clusters relating to post-synaptic membrane potential, post-translational protein modification, protein folding, and molecular chaperones and mitochondrial respiratory function in the EEP vs. SSP comparison. In contrast, none of these clusters are present in the SEP vs. SSP analysis of acute ethanol responses (**Figure 3D**), which did show categories related to actin filament function and small GTPase signal transduction (**Figure 3C**). Similarly, the EES vs. SSS and SES vs. SSS comparisons showed functional dissimilarity with each other and the P2 comparisons for the most part (**Figures 3E,F**) except for the occurrence of clusters relating to molecular chaperone function in the EES vs. SSS comparison, similar to that seen in the P2 sensitization response (**Figure 3D**). Complete details of all functional over-representation studies for these group comparisons are contained in **Supplementary Tables S6**, **S7**. Overall, this gene level functional analysis suggests that ethanol sensitization produces a striking synaptic transcriptome response with changes in expression groups affecting energy production, protein trafficking/folding, and post-synaptic membrane currents.

### Ethanol Sensitization Is Accompanied by Differential Splicing Events

**FIGURE 4:**
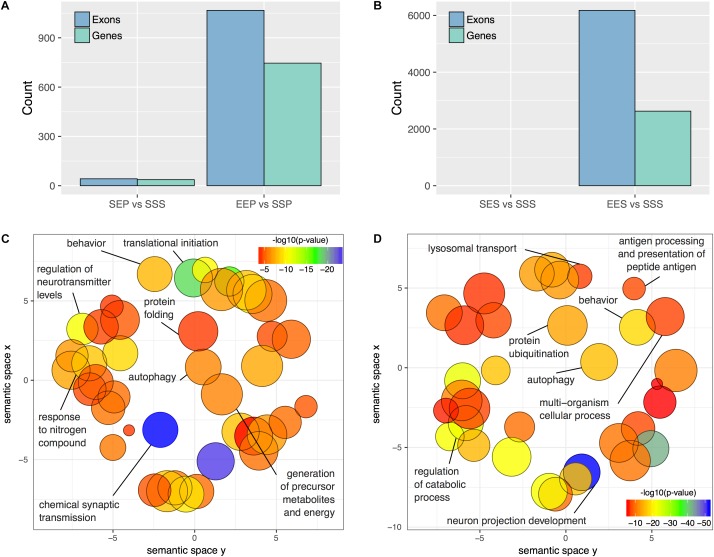
DEU in P2 and S2 fractions following acute ethanol exposure or sensitization. The number of differentially utilized exons (*p*adj < 0.01) and unique genes possessing a minimum of one differentially utilized exon observed in the P2 **(A)** and S2 **(B)** fractions following acute ethanol exposure or sensitization. Gene Ontology reduction plots depicting clustering of top biological processes associated with ethanol sensitization and acute exposure induced DEU are displayed for the P2 **(C)** and S2 **(D)** fractions.

Since differential splicing and transcript utilization are prominent in the nervous system, we performed an exon-level analysis of treatment effects within the P2 and S2 compartments using DEXSeq. We used a more stringent statistical threshold (adjusted *p*-value ≤ 0.01) to defined DEU due to the nearly 30-fold greater number of exons detected (*n* = 356,131; **Supplementary Table S9**) compared to the number of genes detected with edgR (*n* = 11,764; **Supplementary Table S3**). DEXSeq analysis revealed widespread alternative splicing events in the frontal pole S2 and P2 of ethanol sensitized mice. 1067 exons were differentially utilized in the P2 fraction following ethanol sensitization (EEP vs. SSP), representing 746 unique genes (**Figure 4A** and **Supplementary Table S10**). In contrast, only 42 exons representing 36 genes were differentially utilized in the acute ethanol exposure group (SEP vs. SSP; **Figure 4A** and **Supplementary Table S10**). In the somatic fractions of sensitized mice, 6179 exons representing 2627 genes were differentially utilized (EES vs. SSS), whereas no exons passed our statistical threshold in the acute ethanol exposure group (**Figure 4B** and **Supplementary Table S11**).

Functional enrichment analysis of P2 genes affected by ethanol sensitization-induced DEU revealed perturbed Gene Ontology Biological Processes (*p* < 0.01) relevant to translation regulation, mRNA processing, protein stability, and synaptic function (**Figure 4C** and **Supplementary Table S10**). In contrast, Gene Ontology Biological Processes affected by sensitization (*p* < 0.01) in the S2 fraction were primarily involved in catabolism, autophagy, and regulation of cellular morphology (**Figure 4D** and **Supplementary Table S11**). Over-representation analysis was not performed for the acute ethanol exposure groups due to the low level of affected exons.

### RNA Binding Protein Targets Are Enriched in P2 Exons Regulated by Ethanol Sensitization

**FIGURE 5:**
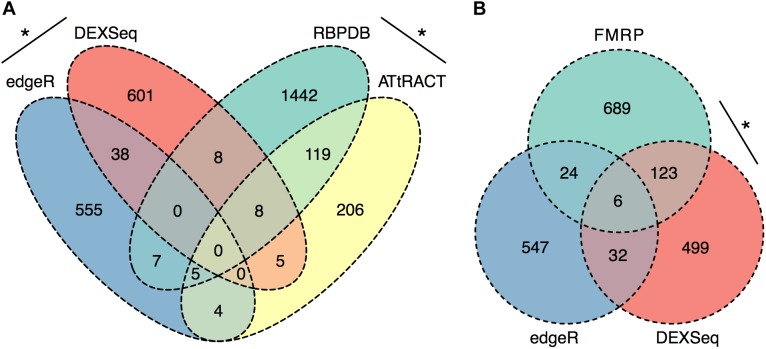
Synapse-Specific DEU is enriched in RNABP targets. Ethanol sensitization altered synaptic exon usage for targets of RNA binding proteins. Ethanol sensitization did not enrich gene expression or differential exon usage **(A)** of RNA binding proteins at synapses. Synaptic DEU but not DGE were enriched for targets of FMRP following ethanol sensitization **(B)** (^∗^*p* < 0.05, Fisher’s exact test).

To further evaluate the RNA processing and translation-related functional categories present in the ethanol sensitization-dependent P2 DEU functional enrichment analysis, the significant P2 DEU and DGE gene lists were analyzed for enrichment in RBPs using two publicly available databases, RBPDB and ATtRACT. To focus more conservatively on synaptic mRNA regulated by ethanol sensitization, we used the intersection of EEP vs. SSP and SSP vs. SSS gene or exon datasets for these analyses. The DGE (**Supplementary Table S4**) and DEU (**Supplementary Table S12**) gene lists showed a modest but significant overlap with each other (OR = 2.3, *p* = 1 × 10^-5^) as did the databases of RBPDB and ATtRACT (OR = 8.8, *p* = 9.6 × 10^-63^; **Figure 5A**). However, the DGE list was not enriched for RNABPs from RBPDB (OR = 0.3, *p* = 1) or ATtRACT (OR = 1, *p* = 0.59) nor was the DEU list enriched for RNABPs from RBPDB (OR = 0.3, *p* = 1) or ATtRACT (OR = 1.3, *p* = 0.22; **Figure 5A**).

The same sensitization-induced synaptic DGE and DEU gene lists were then evaluated for enrichment of RNA targets of a synaptically ubiquitous RNABP, FMRP. FMRP has previously been identified as being involved in ethanol regulation of GABA_B_ receptor membrane abundance (Wolfe et al., 2016). The DGE gene list was not found to be enriched in FMRP targets (OR = 1.4, *p* = 0.07) whereas the DEU gene list showed marked over-representation for FMRP targets (OR = 7.2, *p* = 1.1 × 10^-56^; **Figure 5B**).

Due to the lack of enrichment of RNABPs but over-representation of RNABP targets in the sensitization-induced synaptic DEU gene list, the possibility for novel or known sequence motifs governing RNABP target preference was investigated within the differentially utilized exon bins. Exon bin sequences were supplied to the web-based motif discovery tool MEME and five novel sequence motifs were detected within the exon list having *E*-values ≤ 0.05 (**Table 1** and **Supplementary Table S13**). Of these, four were also found to have high sequence alignment with known RNABP sequence preferences (*E* ≤ 0.05) from the CISRNA-BP database. These findings suggest that a discreet set of RNABPs may regulate synaptic trafficking of ethanol sensitization-responsive transcripts.

**Table 1:** Sequence motif discovery in P2 ethanol sensitization-regulated exons.

Motif	Logo	*E*-value	Similar motifs
1	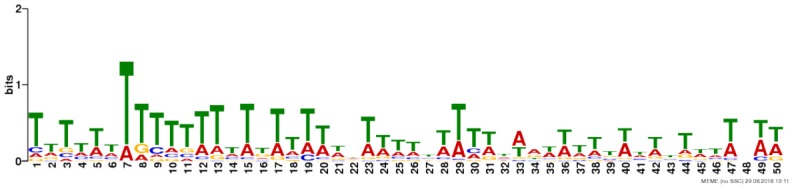	1 × 10^-170^	Gm10110
2	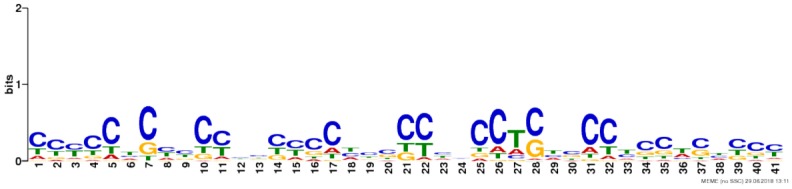	3.6 × 10^-93^	Srsf4
3	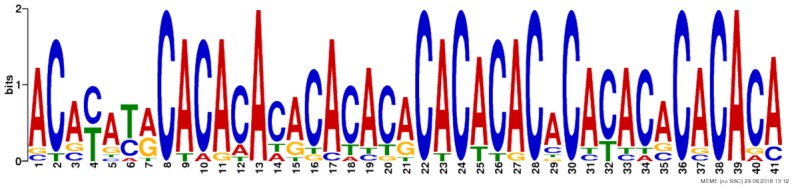	3.8 × 10^-13^	Celf3 Celf4 Hnrpll Rbm38
4	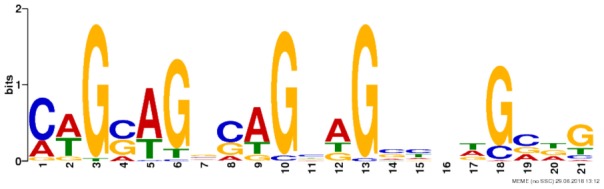	1.2 × 10^-8^	None known
5	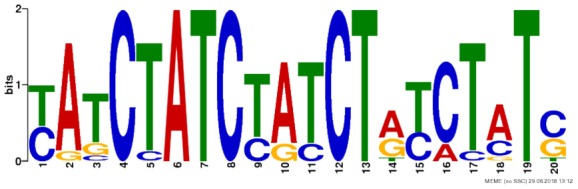	2.5 × 10^-8^	Hnrpdl


## Discussion

The studies contained here provided the first genomic analysis of acute ethanol and ethanol sensitization regulation of the synaptic transcriptome. Using a well-characterized synaptoneurosomal preparation, we validated enrichment of synapse-related mRNA. RNAseq analysis showed that both acute ethanol and ethanol sensitization, a model of behavioral plasticity, produced unique changes in the synaptic transcriptome. In particular, ethanol sensitization produced increased synaptic expression of genes that function in protein synthesis and folding and dendritic structure, among others. We also demonstrated, using an exon-level analysis, a striking preponderance of differential exon utilization occurring following ethanol sensitization. The genes showing DEU with ethanol sensitization were over-represented for targets of specific RBPs, including FMRP. Thus, ethanol sensitization has a major impact on the synaptic transcriptome in both regulation of gene expression and transcript composition. The genes identified here as regulated by ethanol sensitization in the synaptic transcriptome may provide unique understanding of the mechanisms underlying synaptic plasticity contributing to behavioral changes occurring with chronic ethanol exposure.

Neurons are highly specialized polarized cells, whose dendritic and axonal arborizations contain thousands of synapses that function and change individually in response to stimulation (Steward and Levy, 1982; Steward et al., 1998; Wallace et al., 1998). It has been proposed that activity-dependent synaptic plasticity requires the transport and translation of specific mRNA species, creating a unique complement of proteins that are able to function in response to a specific stimulus (Bramham and Wells, 2007). Comparing the somatic and synaptic transcriptomes in response to acute or sensitizing treatments of ethanol, we were able to detect compartmentalized differences in ethanol regulation of gene expression. Through our initial characterization studies, we are confident in our assessment that the differences observed when analyzing the P2 and S2 fractions are a survey of ethanol’s effect on gene expression in distinct subcellular locations. The exact means by which ethanol is exerting its regulation of the synaptic transcriptome has yet to be determined. Conceivably, ethanol could be affecting synaptic transcript abundances through overall modulation of gene expression that could have a global effect on mRNA levels within the cell, and ultimately, through the mere altered availability of transcript, results in changes at the synapse. Our data indicate that this is not an adequate explanation, as we were able to detect distinct gene sets representing different biological function categories in the P2 and S2 fractions. Furthermore, there was a striking lack of overlap between functional categories regulated by acute vs. sensitizing ethanol treatments, despite both assays being done at the same time frame post-ethanol exposure. This is clear evidence of reorganization of the synaptic transcriptome with chronic ethanol exposure.

The trafficking and localization of transcripts to the synapse offers another possible means of regulatory control. Synaptic tagging is a process whereby synaptic activation induces a transient synapse-specific change that allows the synapse to capture mRNA or proteins required for long-term plasticity, which has explicitly been studied for its role in long-term potentiation (Frey and Morris, 1997). The exact physical nature of the synaptic tag has not been absolutely defined, but candidate molecular tags that have been proposed include post-translation modifications to existing synaptic proteins, alterations to protein conformational states, initiation of localized translation or proteolysis, and reorganization of the local cytoskeleton (Martin and Kosik, 2002; Kelleher et al., 2004; Doyle and Kiebler, 2011). All of these mechanisms have the potential of being initiated by signaling events that result from membrane receptor activation. For instance, one pharmacological effect of ethanol is the release of dopamine in the nucleus accumbens, which when acting at D1-like receptors increases activity of adenylyl cyclase, thereby increasing cAMP levels and PKA activity. It has been shown that PKA activation is required for the formation of the synaptic tag (Casadio et al., 1999; Barco et al., 2002). The premise that signaling cascades downstream of ethanol could alter the ability of activated synapses to capture dendritically targeted mRNA requires examination.

Regardless of the exact mechanisms for synaptic localization of mRNA, our data here clearly suggest that differential activation or expression of RBPs by ethanol sensitization may be a major mechanism for restructuring the synaptic transcriptome to produce enhanced locomotor activation following repeated ethanol exposure. Our motif binding overrepresentation analysis of DEU results adds supportive evidence for ethanol sensitization utilizing specific mRNA binding proteins for modulating the synaptic transcriptome by identifying five novel consensus sequences with high similarity to known or predicted RNABP targets. Furthermore, this mechanism is strongly supported by our finding that genes with ethanol sensitization-induced DEU in the synaptic fraction are strongly over-represented for targets of the mRNA binding protein FMRP. FMRP is a known RNA-binding protein involved in mRNA transport and regulation of synaptic protein translation, as well as dendritic spine development (Darnell et al., 2011; Cruz-Martin et al., 2012; Michaelsen-Preusse et al., 2018). Prior studies on ethanol and FMRP have shown that the protein can regulate an acute ethanol-induced alteration in GABA type B receptor (GABABR) dendritic expression (Wolfe et al., 2016). Spencer et al. (2016) also showed that chronic ethanol exposure altered expression of NMDA, Kv4.2, and KChIP3 in hippocampus in an FMRP-dependent fashion, possibly by altering phosphorylation of FMRP and its translational inhibitory properties. Our studies here greatly extend this connection between ethanol, FMRP, and synaptic plasticity. **Figure 5** demonstrates that 20% (129/660; *p* = 1.1 × 10^-56^) of the genes showing ethanol sensitization-induced DEU and enriched in the P2 fraction also overlapped with presumed FMRP target mRNA. This utilization of FMRP targeting by ethanol sensitization clearly implicates this subset of genes in mechanisms of ethanol-induced synaptic plasticity and may have implications for overlap of AUD with other neurological disorders.

Another major finding in these studies is that repeated dosing of ethanol to produce sensitization in D2 males induces substantially more DGE than acute ethanol in both the P2 and S2 fractions. Strikingly, DEU was almost exclusively seen in the ethanol sensitized mice. The bioinformatics analysis of our P2 candidate gene list indicated that transcripts altered in response to repeated ethanol are significantly enriched for biological functions associated with post-synaptic membrane potential, posttranslational protein modifications, protein folding and molecular chaperones, and mitochondrial function. Previously, our laboratory has shown that ethanol regulates transcription and mRNA abundance of molecular chaperones *in vitro* and *in vivo* (Miles et al., 1991, 1994; Kerns et al., 2005). The present study extends these findings by providing evidence that this regulation may be localized or at least occurring at the synapse. Acute ethanol induced significantly fewer expression changes that represented distinct biological categories including actin filament and small GTPase signal transduction. The robust expression response to ethanol sensitization is striking in that some of our prior studies have documented actual habituation of some expression responses (*Sgk1*) to acute ethanol following ethanol sensitization induction (Costin et al., 2013a).

The large expression responses to both acute ethanol and ethanol sensitization with gene-level analysis of our RNAseq data was in striking contrast to our finding that ethanol sensitization alone led to robust alterations of exon usage in both the synaptoneurosome and somatic fractions. Very few exons were differentially utilized following acute ethanol. However, the categories of genes altered by ethanol sensitization either at the gene level or exon utilization show functional overlap with biological processes of RNA translation, RNA processing, and cellular energetics. This functional over-representation is consistent with altered demands on synaptic activity and synaptic protein synthesis with sensitization. However, the striking predominance of exon utilization regulation by sensitization suggests that a form of transcriptional plasticity accompanying the synaptic and behavioral plasticity seen with repeated ethanol exposure. The mechanism(s) for such differential exon utilization may be linked to the need for trafficking mRNA to the synapse. Such a response is suggested by our finding that sensitization-responsive DEU genes were over-represented for FMRP target mRNA, but that at the gene level, sensitization did not evoke an over-representation of FMRP targets in the synaptic transcriptome (data not shown). The mechanism whereby sensitization might alter promoter utilization, splicing or mRNA stability in producing such a robust DEU response at the synapse remains to be determined.

In *a prior* study, Most et al. (2015) reported microarray analysis of expression changes in a synaptoneurosome preparation from amygdala in C57BL/6J mice following prolonged ethanol oral consumption. That study also identified changes relating to protein synthesis in the ethanol-regulated synaptic mRNA. However, there was no clear connection to a form of plasticity in their studies, although progressive ethanol consumption is thought to involve synaptic plasticity. Furthermore, those studies did not involve an exon-level analysis so direct comparison to our results here is not possible. Regardless, Most et al. (2015) did find a much more vigorous ethanol-responsive gene expression regulation in the synaptoneurosome as compared to a total cellular lysate. Their studies thus complement our findings on the dramatic response to ethanol at the level of the synaptic transcriptome. Together, our studies emphasize the importance of analyzing ethanol transcriptional responses at a more precise cellular and subcellular level so as to more clearly identify biological mechanisms and consequences. A minor drawback to both our current studies and those of Most et al. (2015) is the lack of validation of RNAseq results by additional techniques such as RT-PCR or western blot analysis, or preferably, by cellular resolution techniques such as *in situ* hybridization or immunohistochemistry. Such studies were not a major goal of the current report, where we have focused on network- or pathway-level finding rather that single genes. We did provide at least a partial cross validation of our molecular findings in our studies on select candidate genes shown in **Figure 2B** and **Supplementary Table S6**. However, future detailed cellular validation studies are clearly needed.

Using expression analysis, our study is the first to characterize regulation of the synaptic transcriptome by ethanol (or any exogenous drug) in an *in vivo* model of synaptic plasticity. With repeated intermittent exposure to ethanol that resulted in a sensitized response, we observed changes to the complement of mRNA present at the synapse and alterations in the exonic composition of synaptic mRNA that we hypothesize contribute to the development of the behavioral phenotype in D2 mice. The individual genes and functional groups (e.g., molecular chaperones) identified in these studies provide important new information regarding the mechanisms of ethanol-induced synaptic plasticity. Perhaps most importantly, however, our studies have identified that ethanol sensitization uniquely regulates exon utilization at the synapse in a manner that implicates specific RBP targeting, such as by FMRP. Functional analyses will be required to further validate these results with the ultimate goal of disrupting synaptic targeting of specific transcripts or groups of transcripts in order to causally relate this mechanism to synaptic plasticity and modulation of ethanol behaviors.

## Author Contributions

MO’B and MM conceived and designed the study. MO’B conducted primary behavioral studies, synaptoneurosome optimization and isolation, and initial molecular studies. RMW conducted primary RNAseq analysis including detailed exon-level studies and motif analysis. NS and SB performed initial low-level analysis of RNAseq data. JB assisted with electron microscopy studies. AP and RWW provided DBA/2J genomic sequence data to identify SNP modifications to C57BL6/J genome for RNAseq alignments. MO’B, RMW, and MM conducted bioinformatics analyses and prepared primary versions of the manuscript. JW assisted on manuscript design, editing, and interpretation of results. MM provided resources for conducting experiments.

## Conflict of Interest Statement

The authors declare that the research was conducted in the absence of any commercial or financial relationships that could be construed as a potential conflict of interest. The handling Editor declared a shared affiliation, though no other collaboration, with the authors AP and RWW at the time of review.

## Footnotes

1 https://genome.ucsc.edu/cgi-bin/hgTables

2 http://genome.sph.umich.edu/wiki/BamUtil

3 https://www.r-project.org

4 http://toppgene

5 http://shenlab-sinai.github.io/shenlab-sinai/

## Supplementary Material

FIGURE S1 Characterization of the synaptoneurosome preparation. **(A)** Schematic depicting synaptoneurosome preparation. Whole homogenate (WH) processed from pooled frontal pole tissue of four mice was used in the centrifugation/filtration scheme depicted here. The initial pellet (P1) contained cellular debris and nuclei. The supernatant from the initial centrifugation (S1) was filtered and subjected to a second centrifugation. The pellet, P2, was enriched for synaptic elements and dendritically targeted RNA as compared to the supernatant, S2, which contained the remainder of the somatodendritic RNA. **(B)** DAPI staining of synaptoneurosome fractions at 20x magnification indicting that most, if not all the nuclei were removed during the initial centrifugation step to produce the P1 pellet. **(C)** Quantification of immunoblots probing subcellular protein markers across synaptoneurosome fractions (H4 = nuclear; LDH = cytosolic; PSD95 = post-synaptic; SYT = presynaptic).

TABLE S1 Antibody specifications. **(b)** qRT-PCR primer specifications.

TABLE S2 RNA-Seq metrics.

TABLE S3 EdgeR CPM data (gene level).

TABLE S4 EdgeR differential expression results (unfiltered data).

TABLE S5 P2 enriched and S2 enriched genes and ToppGene analysis.

TABLE S6 RNAseq results for control genes.

TABLE S7 EEP_SSP or SEP_SSP regulated genes (RNA-Seq, edgeR, FDR = 0.1), ToppFun functional enrichment analysis summary.

TABLE S8 EES_SSS or SES_SSS regulated genes (RNA-Seq, edgeR, FDR = 0.1), ToppFun functional enrichment analysis summary.

TABLE S9 DEXSeq differential exon usage results (unfiltered data).

TABLE S10 EEP_SSP or SEP_SSP regulated exon genes (RNA-Seq, DEXSeq, *p*adj < 0.01), ToppFun functional enrichment analysis summary.

TABLE S11 EES_SSS or SES_SSS regulated exon genes (RNA-Seq, DEXSeq, *p*adj < 0.01), ToppFun functional enrichment analysis summary.

TABLE S12 Synapse-specific differential exon usage.

TABLE S13 MEME motif count matrices.
