# Supplementary figures and images for: Ethanol-Induced Behavioral Sensitization Alters the Synaptic Transcriptome and Exon Utilization in DBA/2J Mice
Source: Front Genet. 2018 Sep 24;9:402. doi: 10.3389/fgene.2018.00402 (PMC6166094; doi:10.3389/fgene.2018.00402)

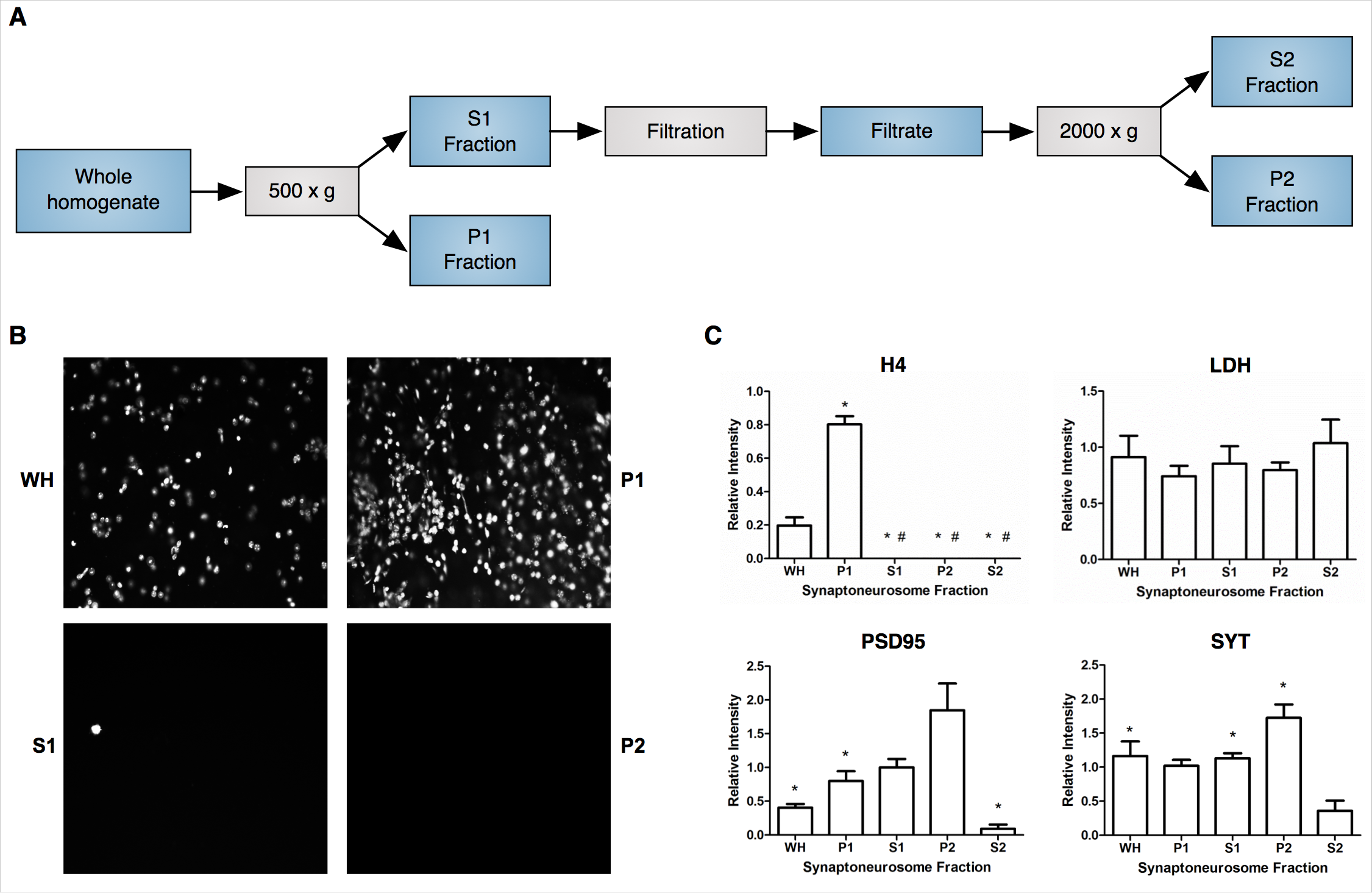

Supplement: FIGURE S1 — Characterization of the synaptoneurosome preparation. (A) Schematic depicting synaptoneurosome preparation. Whole homogenate (WH) processed from pooled frontal pole tissue of four mice was used in the centrifugation/filtration scheme depicted here. The initial pellet (P1) contained cellular debris and nuclei. The supernatant from the initial centrifugation (S1) was filtered and subjected to a second centrifugation. The pellet, P2, was enriched for synaptic elements and dendritically targeted RNA as compared to the supernatant, S2, which contained the remainder of the somatodendritic RNA. (B) DAPI staining of synaptoneurosome fractions at 20x magnification indicting that most, if not all the nuclei were removed during the initial centrifugation step to produce the P1 pellet. (C) Quantification of immunoblots probing subcellular protein markers across synaptoneurosome fractions (H4 = nuclear; LDH = cytosolic; PSD95 = post-synaptic; SYT = presynaptic). [file Image_1.TIFF]
