# Supplementary material for: Ethanol-Induced Behavioral Sensitization Alters the Synaptic Transcriptome and Exon Utilization in DBA/2J Mice
Source: Front Genet. 2018 Sep 24;9:402. doi: 10.3389/fgene.2018.00402 (PMC6166094; doi:10.3389/fgene.2018.00402)
Supplement: TABLE S6 — RNAseq results for control genes. [file Table_6.DOCX]

**Supplemental Table S6: RNAseq Results for Control Genes**

| **Gene** | **AveCPM_SSS** | **AveCPM_SSP** | **Log2_FC_SSP_SSS** | **FDR_SSP_SSS** |
| --- | --- | --- | --- | --- |
| ***Arc*** | 26.57 | 39.52 | 0.57 | 0.01 |
| ***Camk2a*** | 1386.43 | 4743.3 | 1.77 | 8.43E-62 |
| ***Gapdh*** | 12.94 | 10.09 | -0.36 | 0.015 |

RNAseq data are from EdgR analysis results. AveCPM values are from Suppl. Table S4 and are count data normalized per million reads. Log2FC and FDR data are from Suppl. Table S5. Genes portrayed are those used for qRT-PCR validation of synaptoneurosome fractionation (Fig. 2b). Gene *Snrpn* was not detected in RNAseq data.
